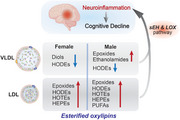# Lipoprotein‐Mediated Esterified Oxylipins Changed in Alzheimer's Disease

**DOI:** 10.1002/alz70856_099173

**Published:** 2025-12-25

**Authors:** Sitong Zhou, Kamil Borkowski, Angela M. Zivkovic, Izumi Maezawa, John W. Newman, Lee‐Way Jin

**Affiliations:** ^1^ University of California Davis Medical Center, Sacramento, CA, USA; ^2^ University of California, Davis, Davis, CA, USA; ^3^ United States Department of Agriculture, Agriculture Research Service, Davis, CA, USA

## Abstract

**Background:**

Inflammation is crucial in Alzheimer's Disease (AD) pathology, with oxylipins – oxidized lipid derivatives of polyunsaturated fatty acids – serving as potent modulators. Particularly, dysregulation of soluble epoxide hydrolase (sEH), which converts pro‐resolving epoxides to pro‐inflammatory diols, has been observed in blood and brain of AD patients. While oxylipins are widely recognized in inflammatory pathways, their transport within plasma lipoproteins is drawing attention, as over 90% of oxylipins circulate in an esterified form within these particles. However, the role of lipoprotein–mediated oxylipin metabolism in AD remains poorly understood.

**Method:**

Plasma low‐density lipoprotein (LDL) and very low‐density lipoprotein (VLDL) fractions were isolated from participants providing samples at the UC Davis Alzheimer's Disease Research Center, including 100 controls, 41 mild cognitive impairment (MCI), and 76 possible AD cases, diagnosed as per NIA‐AA guidelines. Esterified oxylipins were quantified using LC‐MS/MS‐based methods. Aberrant metabolites associations with MCI or AD were analyzed, adjusting for sex, age, BMI and ApoE genotype. Sex‐specific differences were also explored.

**Result:**

Lipoprotein‐associated oxylipin alterations are observed in MCI and AD compared to cognitively normal subjects. Specifically, pro‐inflammatory diols in female LDL decrease with cognitive decline, while pro‐resolving epoxides increase in female LDL and male LDL/VLDL. This inversely aligns with published results showing elevated free diols in AD plasma, suggesting liberation of the esterified oxylipins reservoir into the free oxylipin pool. Oxylipins from lipoxygenase/auto‐oxidative pathways (HODEs, HOTEs and HEPEs) show a gradual increase with cognitive decline. ApoE genotype also appears to moderate these changes, with ApoE3/4 carriers exhibiting elevated epoxides in male VLDL and female LDL, suggesting a reduced circulating anti‐inflammatory tone in cognitive impairment.

**Conclusion:**

Our data reveals significant alterations in lipoprotein‐associated lipid mediators across the spectrum of cognitive decline from MCI to AD, with distinct changes observed in VLDL and LDL fractions. These findings indicate that lipoprotein‐mediated oxylipin dysregulation is sex‐ and ApoE‐ specific, involving sEH and lipoxygenase/auto‐oxidative pathways, highlighting the critical role of inflammation in AD pathology. This supports our hypothesis that lipoprotein composition influences neuroinflammatory processes and could complement the development of plasma biomarkers for AD.